# Prevalence of frailty and its ability to predict in hospital delirium, falls, and 6-month mortality in hospitalized older patients

**DOI:** 10.1186/1471-2318-14-1

**Published:** 2014-01-06

**Authors:** Etienne Joosten, Mathias Demuynck, Elke Detroyer, Koen Milisen

**Affiliations:** 1 Department of Internal Medicine, Division of Geriatric Medicine, University Hospitals, Leuven, Belgium; 2 Health Services and Nursing Research, KU Leuven, Leuven, Belgium; 3 Department of Health Service, Katholieke Hogeschool Limburg, Hasselt, Belgium

**Keywords:** Risk assessment, Elderly, Cardiovascular Health Study (CHS) frailty index, Study of Osteoporotic Fracture (SOF) frailty index, Delirium, Falls, Mortality

## Abstract

**Background:**

The prevalence and significance of frailty are seldom studied in hospitalized patients. Aim of this study is to evaluate the prevalence of frailty and to determine the extent that frailty predicts delirium, falls and mortality in hospitalized older patients.

**Methods:**

In a prospective study of 220 older patients, frailty was determined using the Cardiovascular Health Study (CHS) and the Study of Osteoporotic Fracture (SOF) frailty index. Patients were classified as nonfrail, prefrail, and frail, according to the specific criteria. Covariates included clinical and laboratory parameters. Outcome variables included in hospital delirium and falls, and 6-month mortality.

**Results:**

The CHS frailty index was available in all 220 patients, of which 1.5% were classified as being nonfrail, 58.5% as prefrail, and 40% as frail. The SOF frailty index was available in 204 patients, of which 16% were classified as being nonfrail, 51.5% as prefrail, and 32.5% as frail. Frailty, as identified by the CHS and SOF indexes, was a significant risk factor for 6-month mortality. However, after adjustment for multiple risk factors, frailty remained a strong independent risk factor only for the model with the CHS index (OR 4.7, 95% CI 1.7-12.8). Frailty (identified by CHS and SOF indexes) was not found to be a risk factor for delirium or falls.

**Conclusions:**

Frailty, as measured by the CHS index, is an independent risk factor for 6-month mortality. The CHS and the SOF indexes have limited value as risk assessment tools for specific geriatric syndromes (e.g., falls and delirium) in hospitalized older patients.

## Background

Frailty in older subjects has been defined as a state of decreased functional reserve and resistance to stressors that are associated with a high prevalence of adverse health outcomes, such as poor functional and cognitive status, falls, institutionalization, and mortality [1,2]. Although identifying and measuring frailty is one of the great challenges in geriatric medicine, there is no agreement on a single operational definition for clinical use [3], making it difficult to compare and interpret different research results on frailty.

The prevalence of frailty varies widely depending on its definitions, patient selection, and socioeconomic factors like education. In a European study involving 10 different countries, frailty prevalence was 4.1% in non-hospitalized subjects aged 50–65 years and 17% in subjects aged 65 years and older [4]. Collard et al. reported widely differing prevalences of frailty, ranging between 4.0% and 59.1% in community-dwelling elderly adults, with an overall weighted prevalence of 10.7% for frailty and 41.6% for prefrailty [5]. In older hospitalized patients, the frailty prevalence varied from 27% to 80% [6-8].

One of the most widely used operational definitions of frailty is based on data from the Cardiovascular Health Study (CHS) [1]. Another instrument for measuring frailty is the Study of Osteoporotic Fractures (SOF) frailty index [9]. Some studies have compared the CHS and SOF indexes and have found that both were good predictors of hospitalization, falls, fracture, and death in *non*-hospitalized older adults [9,10]. Numerous other frailty tools have been validated and each of these has its own strengths and weaknesses [11,12]. Frailty has been linked to the development and progression of many age-related diseases and syndromes, mostly driven by chronic inflammatory processes associated with aging [13-17]. Little is known about the significance of frailty as a predictor for comorbidities and as a risk factor for specific geriatric syndromes in hospitalized older patients. The aim of this study was to evaluate the prevalence of frailty in hospitalized older patients, as determined by the CHS and SOF indexes, and to determine the extent that frailty can predict delirium and falls during hospitalization, and mortality 6 months after discharge. Although we are aware that there are other well validated frailty indexes, the CHS and SOF indexes were chosen because of their simplicity, conciseness and literature-based evidence.

This study was part of a broader ongoing investigation on the effect of a delirium e-learning program on delirium detection and different patient outcomes in hospitalized older patients.

## Methods

### Patients

In a prospective study, 511 consecutive older patients aged 70 years and older were invited to participate. All patients were admitted to the acute geriatric ward of a tertiary care hospital. The study took place during two 4-month periods separated by 3 months, during which no measurements were made. Ninety-five percent of these patients were first admitted to the emergency department.

### Definition of frailty

In the first step, frailty was assessed 24 hours after admission according to the five CHS criteria [see ref [1] for definition and cut-off points]. Criterion 1 was unintentional weight loss of >5 kg during the last year or a BMI of <18.5. Criterion 2 was slow walking speed during a 4.5 m walk. For men, walking speed was considered to be “slow” if it took ≥7 or ≥6 seconds to walk 4.5 m for men with a height of ≤173 cm or >173 cm, respectively. For women, walking speed was considered to be “slow” if it took ≥7 or ≥6 seconds for women with a height of ≤159 cm or >159 cm, respectively. The use of a walking aid was acceptable. Criterion 3 was reduced energy, which was determined by the patient answering “no” to the question, “Do you feel full of energy?” Criterion 4 was low physical activity level, which was determined by the patient answering “less” to the question, “Are you more, less, or equally active compared to men and women of your age?” Criterion 5 was grip strength, which was adjusted for gender and BMI. Grip strength was measured with a Jamar hydraulic hand dynamometer. The best result of three attempts was taken as the final result. For men, low grip strength was defined as ≤29 kg for men with a BMI of ≤24, ≤30 kg for men with a BMI between 24.1 and 28 and ≤32 kg for men with a BMI of >28. For women, low grip strength was defined as ≤17 kg for women with a BMI of ≤23, ≤17.3 kg for women with a BMI between 23.1 and 26, ≤18 kg for women with a BMI between 26.1 and 29, and ≤21 kg for women with a BMI of >29.

Patients who exhibited three or more of these criteria, even if results from fewer than five were available, were considered to be frail. Those displaying one or two criteria were considered to be prefrail. Patients displaying none of the five were defined as nonfrail, if results were available for all five criteria.

In the second step, the SOF frailty index tool was employed in patients having a complete evaluation with the CHS frailty index [9]. The following items were investigated: (1) unintentional weight loss of >5 kg during the last year or a BMI of <18.5; (2) inability to rise five times from a chair without using arms and; (3) answering “no” to the question, “Do you feel full of energy?” Patients displaying two or three of these criteria were considered to be frail, if the results of at least two criteria were available. Patients were considered to be prefrail and nonfrail if they displayed one or no criterion, respectively, and only if the results of the three criteria were available.

### Other variables

All patients were examined by their attending geriatrician, who was responsible for the medical follow-up and medical data. For each patient, the number of comorbidities and the number of medications taken at home were abstracted from the medical records or requested from a family member, a caregiver, or the patient’s family physician. Comorbidities were slightly modified from the Charlson Comorbidity Score [18]. Because some of the diseases included in this index are less representative for our population (i.e. there were no patients with AIDS, leukaemia, malignant lymphoma and there are no strict criteria to differentiate between mild and moderate vs. severe liver diseases), the following comorbidities were retained: myocardial infarct, congestive heart failure, peripheral vascular disease, cerebrovascular disease, dementia, chronic pulmonary disease, connective tissue disease, gastric ulcer disease, liver disease, diabetes, hemiplegia, renal failure and any cancer including hematological malignancies.

Cognitive status was evaluated using the 12-item Mini-Mental State Examination (MMSE) [19]. This is a short version of the original MMSE with a maximum score of 12 but preserves the diagnostic properties of the original MMSE. Education was classified into three categories (<8 years, between 8 and 12 years, >12 years). Functional status during the last 2 weeks before hospitalization was assessed by the research nurses 24 hours after admission using the Katz Index [20]. This included the following six activities of daily living: bathing, dressing, feeding, continence, transfer, and toileting. Each was scored as 0 (independent), 1 (partly dependent), or 2 (dependent), resulting in a total score between 0 to 12. Higher scores indicate greater dependency. Depression was scored 24 hours after admission with the 10-item Geriatric Depression Scale [21] (scale from 0 to 10), with higher scores indicating more severe symptoms. Delirium was diagnosed during hospitalization according to the confusion assessment method (CAM) algorithm: acute onset and fluctuation, inattention, disorganized thinking, and/or altered level of consciousness [22]. Delirium was evaluated on the first, third, fifth, seventh day after admission and on the day before discharge. In patients with a hospitalization of more than seven days, the CAM was further assessed weekly. If the patient had delirium on one of the measurement points, the delirious status was followed up daily until a negative CAM score was obtained. Patients who scored positive for at least 1 CAM assessment were diagnosed with delirium. A fall was defined as an unexpected event in which the patient comes to rest on the ground, floor, or lower level. Patients experiencing one or more falls during hospitalization, as documented by the attending nurse, were categorized as fallers.

Six research nurses measured the frailty indicators and performed the functional and cognitive assessments. They were trained in advance by two experienced research investigators (ED and KM) according to criteria set in the manuals of MMSE [19] and CAM [22] including evaluation of four clinical cases and follow-up discussions,. Interrater reliability for CAM (i.e. agreement of CAM scoring for each research nurse compared with CAM scoring of one of the investigators (ED), and calculated two by two in a random sample of 18 paired observations of enrolled patients) was κ = 1.00. Interrater reliability for the frailty indexes was not tested.

### Mortality

All deaths during hospitalization were recorded. Patients who were discharged from the hospital; their family members or proxies; or the administrator, in case of institutionalization, were contacted by phone by one of the research nurses after 6 months to determine whether the patient had died during this period.

### Laboratory analysis of blood

Blood samples were obtained on admission. A complete blood count was determined using an XE-5000 automated blood counter (Sysmex, Kobe, Japan) and before the administration of packed cell transfusion, if necessary. Anemia was defined according to WHO criteria: for men, a hemoglobin level of <13 g/dL; and for women, a hemoglobin level of <12 g/dL. Severe anemia was defined as a hemoglobin level of <10 g/dl for both men and women, and moderate anemia was defined as a hemoglobin level between 10 and 12 g/dl for women and 10 and 13 g/dl for men. C-reactive protein (CRP) and creatinine levels were analyzed on a Roche/Hitachi Modular P800, estimated glomerular filtration rate (eGFR) was calculated using the Modification of Diet in Renal Disease equation [23].

### Ethics

The Medical ethics committee of the Leuven University Hospitals approved the study and informed (proxy) consent was obtained for each participant before inclusion.

### Statistical methods

Data were analyzed using SPSS version 20 (IBM Corp., Armonk, NY). The Kolmogorov-Smirnov test was used to investigate the normal distribution of the data. Comparison between two groups was carried out by using the Student’s t-test or the Mann–Whitney test, depending on the distribution of the data. The Chi-square or Fischer’s exact test was used for categorical variables. Comparison of different groups was carried out by using the analysis of variance test with a Bonferroni correction for multiple tests between the different groups. Cohen’s kappa was used to measure the agreement between the two frailty indexes [24]. Multiple logistic regression analyses were conducted to evaluate the association between delirium, falls, and 6-month mortality as the dependent variables and the CHS and SOF frailty indexes as the principal variable of interest. We controlled for other potential confounding variables such as age, gender, number of comorbidities, activities of daily living, cognitive impairment, main diagnosis, hemoglobin, depression, and eGFR. P values <0.05 were considered to be statistically significant.

## Results

Of the 511 eligible patients, a total of 250 patients were excluded because of various reasons: patients declined to participate (n = 80); dropped out of the study (n = 27); terminally ill (n = 3); non-Dutch speaking (n = 6); younger than 70 years old (n = 1); impossible to converse minimally (n = 66); severe hearing or visual problems (n = 18); isolation due to acute infectious diseases (n = 9); very poor health condition (n = 22); readmission during the study period (n = 6); discharged or death within 24 hours after admission (n = 12). Of the remaining 261 patients, another 41 were excluded because CHS frailty index data were incomplete, preventing correct interpretation. Data were incomplete, because of limited patient cooperation during the assessment sessions. Hence, the final sample comprised 220 patients.

The main diagnosis on admission for the 220 patients included in our study were infectious diseases (26%), falls-fractures-osteoporosis (17%), gastrointestinal diseases (14%), heart failure and respiratory insufficiency (12%), neuropsychiatric diseases (9%) and cancer (5%). Basic patient characteristics are presented in Table 1, organized according to the two frailty indexes. The CHS index was available in 220 patients, but only 3 (1.5%) were considered as nonfrail, 129 (58.5%) as prefrail, and 88 (40%) as frail. Because there were only 3 nonfrail patients, a number not allowing reliable statistical comparisons, we combined the nonfrail and prefrail patients into one group and denoted this group the “nonfrail/prefrail” group. Thus, 88 frail patients (40%) and 132 nonfrail/prefrail patients (60%) were considered for statistical analysis. The SOF index was available in 204 of these patients, of which 32 (16%) were classified as nonfrail, 104 (51.5%) as prefrail, and 66 (32.5%) as frail.

**Table 1:** Patient characteristics and laboratory and clinical data of patients assessed with the CHS and SOF frailty index*

	**CHS frailty index (N = 220 patients)**			**SOF index (N = 204 patients)**			
	**Nonfrail and prefrail (n = 132)**	**Frail (n = 88)**	**P**	**Nonfrail (n = 32)**	**Prefrail (n = 106)**	**Frail (n = 66)**	**p**
Age (y), mean ± SD	83.7 ± 4.8	83.3 ± 5.4	0.58	83.1 ± 5.2	83.8 ± 5.1	83.5 ± 5.1	0.77
Female, n (%)	81 (62)	45 (51)	0.1	15 (47)	64 (60)	38 (58)	0.39
Number of comorbidities, mean ± SD	2.33 ± 1.5	3.4 ± 2	<0.001	2 ± 1.3^a^	2.4 ± 1.4	3.1 ± 1.8	0.005
Number of medications taken at home, mean ± SD	7.5 ± 3.5	8.9 ± 3.5	0.005	7 ± 3.9^b^	7.7 ± 3.1	8.9 ± 3.6	0.012
Hemoglobin, g/dl, mean ± SD	12.3 (2.1)	11.7 (2.1)	0.07	12.7 ± 2.1	12.1 ± 2.0	11.8 ± 2.0	0.09
Hemoglobin < 10, n (%)	19 (15)	18 (20)	0.32	4 (13)	16 (15)	13 (20)	0.36
Hemoglobin ≥10 and <12 (F) or < 13 (M), n(%)	39 (30)	30 (34)		6 (19)	35 (33)	21 (32)	
Hemoglobin ≥12 (F) or ≥ 13 (M), n (%)	73 (55)	41 (46)		22 (68)	55 (53)	32 (48)	
C-reactive protein (mg/L), mean ± SD	43.6 ± 74	45 ± 63	0.12	51 ± 84	45.5 ± 77	42 ± 61	0.68
eGFR (ml/min), mean ± SD	54.6 ± 22	49.9 ± 24.6	0.16	59.2 ± 18	54.4 ± 23	49.2 ± 24	0.12
Education							
Low (<15 y), n (%)	52 (39.4)	36 (40.1)	0.78	14 (43.8)	43 (40.5)	26 (39.4)	0.9
Moderate (12–18 y), n (%)	66 (50)	41 (46.5)		15 (46.9)	52 (49)	32 (48.5)	
High (≥18 y), n (%)	14 (10.6)	11 (12.5)		3 (9.3)	11 (10.4)	8 (12.1)	
MMSE short form, mean ± SD (range 0–12)	8.5 ± 3.0	8.1 ± 3.1	0.2	9.2 ± 2.9	8.2 ± 3.1	8.8 ± 2.5	0.12
ADL, mean ± SD	2.6 ± 3.0	4.5 ± 3.0	<0.001	1.1 ± 1.7^c^	3.3 ± 3.1	4.1 ± 3.1	<0.001
GDS, mean ± SD	2.7 ± 2.2	4.4 ± 2.6	<0.001	2.5 ± 2.1	3.0 ± 2.4	4.2 ± 2.6^d^	0.001
Patients with delirium during hospitalization, n (%)	14 (10.6)	10 (11.4)	0.86	2 (6.3)	12 (11.3)	6 (9.1)	0.68
Patients with ≥ 1 fall during hospitalization, n (%)	10 (7.6)	8 (9.1)	0.7	0 (0)	12 (11.3)	5 (7.6)	0.12
Length of stay, days, mean ± SD	15 ± 11.6	17.4 ± 13.1	0.17	12.2 ± 8.7	15.5 ± 11.6	17.9 ± 13.2	0.08
Mortality during hospitalization, n (%)	1 (0.8)	9 (10.2)	0.001	0 (0)	3 (2.8)	7 (10.6)	0.02
Morality 6 months after hospitalization^e^, n (%)	7/127 (5.5)	23/77 (30)	<0.001	0/31 (0)	12/99 (12)	13/59 (22)	0.01

*Abbreviations*: *SD* standard deviation, *M* male, *F* female, *CHS* Cardiovascular Health Study, *SOF* Study of Osteoporotic Fracture, *eGFR* estimated glomerular filtration rate, *ADL* activities of daily living, *MMSE* Mini-Mental State Examination, *GDS* Geriatric Depression Scale.

*A total of 220 patients were included. Of the 220 patients who had complete CHS data, 204 had complete SOF data.

^a^Nonfrail patients significantly different from frail patients (p = 0.006 after Bonferroni correction).

^b^Nonfrail patients significantly different from frail patients (p = 0.03 after Bonferroni correction).

^c^Nonfrail patients significantly different from prefrail (p = 0.001 after Bonferroni correction) and frail patients (p < 0.001 after Bonferroni correction).

^d^Frail patients significantly different from prefrail (p = 0.01 after Bonferroni correction) and nonfrail(p = 0.005 after Bonferroni correction) patients.

^e^Data for 6-month mortality were available for 204 patients assessed with the CHS index and 189 patients assessed with the SOF index.

Frail patients had the most comorbidities and were prescribed the most medications. When assessed with the CHS index, there was a tendency towards significantly lower hemoglobin levels in frail as compared to nonfrail/prefrail patients (p = 0.07). By contrast, when assessed with the SOF index, frail, prefrail, and nonfrail patients had comparable mean hemoglobin levels (p = 0.09). Prevalence of moderate and severe anemia, serum CRP levels, and eGFR levels were comparable among all frailty categories for both the CHS and SOF indexes.

Education level, cognitive functioning, and length of stay were similar between the groups. According to both indexes, worse functional capacity, depression score, and mortality were significantly associated with frailty. With regard to assessing falls, the SOF index identified zero fallers in the nonfrail group, 12 fallers in the prefrail group, and 5 fallers in the frail group. These findings, however, were not statistically significant. Although with the CHS index there was no difference in the number of fallers in the nonfrail/prefrail and frail groups of patients, it is noteworthy that none of the 3 patients initially classified as nonfrail according to the three original CHS classification groups fell during hospitalization (data not shown).

Table 2 shows the number of patients completing the different items of the CHS and SOF indexes, and Table 3 shows the agreement between both frailty indexes. As could be expected, items related to a physical task, such as walking speed, grip strength, and rising from a chair, could not be scored in every patient (Table 2). Table 3 shows the classification of the frailty components into 2 (nonfrail/prefrail versus frail) and 3 (nonfrail, prefrail, frail) groups using the CHS and SOF frailty indexes, respectively, for the 204 patients who were assessed with both indexes. The frailty status classification of the two groups (nonfrail/prefrail versus frail) and the three groups (nonfrail, prefrail, frail) were concordant in 173 (85%, kappa = 0.67) and 145 (71%, kappa = 0.49), respectively.

**Table 2:** Items completed by patients assessed with the CHS and the SOF frailty index*

	**CHS index (N = 220 patients)**		**SOF index (N = 204 patients)**	
	**Total number**	**Positive**	**Total number**	**Positive**
**CHS items**				
Weight loss	220	62		
Reduced energy level	220	35		
Reduced physical activity	220	69		
Slow walking speed	200	187		
Reduced grip strength	209	158		
**SOF items**				
Weight loss			220	62
Inability to rise 5 times from a chair			203	159
Reduced energy level			220	35

*Abbreviations*: *CHS* Cardiovascular Health Study, *SOF* Study of Osteoporotic Fracture.

*For each frailty index, the number of a specific item can be lower than the total number of patients available for assessment because not all 5 (CHS index) or 3 (SOF index) items need to be present for evaluating the frailty status (see text).

**Table 3:** Agreement between the CHS and the SOF frailty index in 204 patients assessed with both frailty indexes

	**CHS index**			
	**Nonfrail**	**Prefrail**	**Frail**	**Total**
SOF index				
Nonfrail	3	28	1	32
Prefrail	0	84	22	106
Frail	0	8	58	66
Total	3	120	81	204
Cohen’s kappa: 0.49.				
	**CHS index**			
	**Nonfrail and prefrail**		**Frail**	**Total**
SOF index				
Nonfrail and prefrail	115		23	138
Frail	8		58	66
Total	123		81	204
Cohen’s kappa: 0.67.				

*Abbreviations*: *CHS* Cardiovascular Health Study, *SOF* Study of Osteoporotic Fracture.

Table 4 shows the unadjusted and adjusted odds ratios (95% confidence intervals) for the association between the frailty indexes (nonfrail/prefrail versus frail for both indexes) and delirium, falls, and 6-month mortality. Delirium was found in 24 of the 220 patients and 18 fell at least once during hospitalization (Table 1). It is remarkable that 2 out of the 24 patients with delirium but also 16 out of the 196 patients without delirium experienced at least 1 fall during hospitalization (p = 0.99). In the unadjusted and adjusted logistic regression models, frailty was not found to be a risk factor for delirium or falls (Table 4). Ten patients died during hospitalization, and mortality was significantly higher in frail patients (Table 1).

**Table 4:** Prediction of delirium and falls during hospitalization and 6-month mortality according to the CHS and the SOF frailty index

	**CHS index**	**SOF index**
	**OR (95% CI)**	**OR (95% CI)**
Delirium		
Unadjusted		
Nonfrail/prefrail	1	1
Frail	1.08 (0.45-2.5)	0.88 (0.32-2.4)
Adjusted^a^		
Nonfrail/prefrail	1	1
Frail	0.64 (0.25-2.08)	0.81 (0.21-3.2)
Falls in hospital		
Unadjusted		
Nonfrail/prefrail	1	1
Frail	1.22 (0.46-3.22)	1.16 (0.39-3.45)
Adjusted^a^		
Nonfrail/prefrail	1	1
Frail	0.94 (0.31-2.91)	0.71 (0.21-2.4)
6-month mortality		
Unadjusted		
Nonfrail/prefrail	1	1
Frail	7.32 (2.95-18)	2.75 (1.17-6.5)
Adjusted^a^		
Nonfrail/prefrail	1	1
Frail	4.68 (1.7-12.8)	1.97 (0.75-5.2)

*Abbreviations*: *CI* confidence interval, *OR* odds ratio, *CHS* Cardiovascular Health Study, *SOF* Study of Osteoporotic Fracture.

^a^Adjusted for age, sex, education, number of comorbidities, activities of daily living, cognitive impairment, main diagnosis at admission, hemoglobin, depression, and estimated glomerular filtration rate.

Of the 210 patients who were discharged, 204 patients were assessed with the CHS index. Of these, 30 died within 6 months and 189 were also assessed with the SOF index. Of these 189 patients, 25 patients died (Table 1). Frailty, as identified using the CHS and the SOF indexes, was a significant risk factor for 6-month mortality. After adjustment for multiple risk factors, frailty remained a strong independent risk factor only for the model with the CHS index (OR 4.7, 95% CI 1.7-12.8) (Table 4).

## Discussion

Our results demonstrate that frailty is common in this population. Using the CHS and SOF frailty indexes, we found that 40.5% and 32% of the patients were frail, respectively. It is remarkable that only 1.5% and 16% of the patients assessed according to the CHS and the SOF indexes, respectively, were diagnosed as being nonfrail. Both indexes had limited utility in their ability to discriminate among the different outcome measures of this study. Because the SOF index was performed in those patients who completed the CHS model, it is not surprising that the agreement between the CHS and SOF indexes was moderate (Cohen’s kappa 0.49, frail versus prefrail versus nonfrail ) to good (Cohen’s kappa 0.67, frail versus nonfrail/prefrail) [24]. These criteria are arbitrary and one would perhaps expect even a better agreement (i.e. higher kappa values). However, the values found might be explained by the fact that the number but also the clinical significance of the items in both scales are different (5 of which 2 are related to a physical task for the CHS versus 3 of which 1 is related to a physical task for the SOF index). Disease burden, serum CRP levels, eGFR, anemia, education, and cognitive status were not associated with frailty and frailty was not a significant risk factor for in-hospital delirium and falls. Unlike the SOF index (being a predictor only in the univariate analysis and the unadjusted model), frailty as measured with the CHS index was an independent risk factor for 6-month mortality.

The CHS as well as the SOF index require objective measures of physical function with the focus largely on the musculoskeletal system and their results may preferentially identify those hospitalized patients with a severe acute illness rather than being frail. On the basis that frailty is a state of vulnerability to poor resolution of homeostasis following a stressor event, it is likely that older persons, hospitalized for an acute illness such as pneumonia or cardiac ischemia, would be identified as frail using these performance based measures. This is supported by the fact that the results of the items such as grip strength, walking speed and ability to rise from a chair are abnormal in the majority of the participants.

There is an extensive body of literature about frailty assessment instruments and their ability to accurately measure frailty. A thorough review of the pros and cons of different frailty indexes is beyond the scope of this study and can be found elsewhere [12,25-27]. Most epidemiological data are based on studies in *non*-hospitalized older persons. In a recent systematic review, the prevalence of frailty in community-dwelling elderly subjects varied between 4% and 59% [5]. Many age-related diseases and geriatric syndromes are more prevalent in frail older persons than in the nonfrail [1,7,9,14-16,28-39] but most of this work was done in non-hospitalized older persons.

Frailty prevalence data also vary widely in hospitalized patients. Hubbard et al. compared three frailty tools in three groups (independent, day hospital, continuing care) of older patients [25]. The three frailty scores were each significantly different across the three groups, and according to the CHS index, 100% of the continuing care patients and 72.5% of the day hospital patients were defined as frail, respectively [25]. In another study, 36% of the hospitalized older patients were found to be frail using the Reported Edmonton Frail Score (REFS) [40]. Furthermore, Wou et al. demonstrated that 66.4% and 17.9% of the patients in an acute care setting were assessed as frail with the SOF and CHS index, respectively [41]. A possible explanation for these disparities might be the differences in methodologies used to measure frailty. For instance, the REFS score used in the study of Hilmer et al. [40] contains 9 items (including cognition, social support, medication use and self-reported performance 2 weeks ago as a surrogate for the ‘get up and go’), and these items are very different from those used in the SOF and CHS indexes. Also, these divergent results can partially be explained by selection bias. In our hospital for instance, more than 95% of the geriatric patient population is admitted to the emergency department before they are referred to the geriatric hospitalization ward. Those who are more vulnerable and suffer from multimorbidity are mainly referred to the geriatric ward, while the more independent and less vulnerable elderly patients with a single issue, or with only a few organ dysfunctions, are referred to organ-specific wards such as cardiology, pulmonology, neurology, etc.

Some comments need further explanation and discussion. There is no gold standard frailty test, and numerous tests have been described in the literature that differs substantially in the way they operationalize the frailty concept. As a consequence, it is possible that assessing frailty with another index could change our results. The frailty status of older patients is a dynamic process, with frequent transitions into short periods of time in which they are more or less frail [42]. It is possible that a hospitalized patient with a very limited exercise tolerance or severe mobility impairment due to acute heart failure would initially be assessed as frail based for instance on a slow walking speed according to one of the CHS criteria but with a remarkable recovery after a short period of adequate therapy.

Although frailty as identified by the CHS model was an independent risk factor for mortality, a limitation of our study was the absence of an illness severity measure for which could not be corrected in the multivariate analysis and this might have biased our results. However, number of co-morbidities and ADL score as measures for functional decline can be regarded as proxy measures for illness severity as was corrected for in the logistic regression model.

As opposite to our results, Eeles et al found a strong association between delirium and frailty [16]. Their results can possibly be explained by the fact that an index of accumulated deficits to measure frailty as used in their study, and containing 33 items (versus 5 and 3 items in the CHS and SOF, respectively) could be a more sensitive test for this purpose. Also in contrast to our findings, Ensrud et al demonstrated that frailty is an independent predictor for recurrent falls in a large cohort of community-dwelling older women [43]. A possible explanation to this incongruous finding may be the fact that hospitalized people assessed as frail in our study do not fall more frequently as they are too weak or too sick to get up and walk and are kept under close surveillance during their hospital stay. In addition to this, the outcome ‘falls’ as documented by the attending nurse, might have led to under- reporting and as a consequence this might have biased our findings. However, we believe this unlikely since all nurses were extensively trained (as part of their standard in-hospital training) in the use of the hospital’s standardized fall incident report form, and the occurrence of fallers was in line with the results of a previous multicenter study we did [44].

Assessment of frailty in hospitalized patients is a time-consuming activity and is exhausting for the patients, which explains the large patient dropout rate. We are aware that the number of patients in our study is limited, which is mainly due to a significant dropout (e.g. 250 out of the 511 eligible patients) and lack of cooperation. As a consequence, the largest part of the excluded subjects was most probably frail and this may have biased our findings. A substantial number of trained researchers are needed to obtain the requested data, and as a consequence, this compromises the feasibility of this approach as a routine clinical investigation in daily practice in a geriatric ward. Furthermore, in a clinical geriatric setting it is difficult to include a much larger number of patients due to conditions related to the high vulnerability of these patients. As a consequence, a simple and reliable frailty tool would be more appropriate [25], but it is unclear to what extent interventions aimed at reducing the prevalence and severity of frailty are effective in reducing adverse outcomes in hospitalized elderly [2].

Finally, due to a very low number of nonfrail patients according to the CHS model, we combined nonfrail and prefrail patients into one group and therefore, this limits a full comparison with the SOF model for which the original 3 groups were used.

## Conclusion

Older hospitalized patients are vulnerable by nature, and only a minority are likely to be nonfrail. Frailty as measured by the CHS index is an independent risk factor for 6-month mortality, but both the CHS and the SOF index have limited value as risk assessment tools for specific geriatric outcomes, such as falls and delirium in hospitalized older patients. Further research is needed to investigate whether or not the study of frailty in acutely hospitalized older patients should be pursued and which instruments are considered appropriate in this setting.

## Abbreviations

CHS: Cardiovascular health study; SOF: Study of osteoporotic facture, BMI, body mass index.

## Competing interests

The authors declare that they have no competing interests.

## Authors’ contributions

Study concept and design: EJ, ED and KM. Information concerning laboratory data and clinical diagnosis: EJ, MD and ED. Acquisition of data: MD and ED. Data analysis and interpretation: EJ, MD and KM. Drafting of the manuscript: EJ and KM. Editing and reviewing the final manuscript: EJ, MD, ED and KM. All authors read and approved the final manuscript.

## Pre-publication history

The pre-publication history for this paper can be accessed here:

http://www.biomedcentral.com/1471-2318/14/1/prepub
